# Immune thrombocytopenia in adolescents and young adults

**DOI:** 10.3389/fmed.2025.1553936

**Published:** 2025-03-26

**Authors:** Alexandra Schifferli

**Affiliations:** Department of Hematology/Oncology, University Children’s Hospital Basel, Basel, Switzerland

**Keywords:** ITP, AYAs, secondary ITP, chronic ITP, sustained remission

## Abstract

Previous guidelines for the treatment of immune thrombocytopenia (ITP) have traditionally focused on a dichotomy between pediatric and adult ITP. Adolescents and young adults (AYAs) do not neatly fit into either the pediatric or adult ITP group. A deeper understanding of ITP’s natural history, risk factors for chronicity, and outcomes in AYAs is a crucial first step toward developing tailored treatment algorithms. Such data could form the basis for recommendations targeting this underrepresented yet clinically distinct population. Ultimately, age-adapted trials may improve long-term outcomes, reduce toxicity, and enhance quality of life for AYAs with ITP. The AYAs collaboration—drawing on data from the Pediatric and Adult Registry on Chronic ITP (PARC-ITP), Registre Midi- Pyrénéen-France (CARMEN-France) adult registry in Toulouse, and the National Prospective Cohort for Children with Chronic Autoimmune Cytopenia (OBS’CEREVANCE) in Bordeaux, France—aims to address the information gap in AYAs with ITP. To date, four analyses have been undertaken (using data from 2004 to 2021), each addressing the major clinical aspects of ITP in patients aged 12–25 years: (1) newly diagnosed ITP, (2) chronic disease, (3) refractory courses, and (4) secondary (sITP) forms.

## Introduction

Previous guidelines for the treatment of immune thrombocytopenia (ITP) have traditionally focused on a dichotomy between pediatric and adult ITP. Pediatric ITP is typically acute and self-limiting, with a very low risk of life-threatening bleeding events. In contrast, adult ITP usually follows a chronic course and is associated with a higher risk of severe bleeding, particularly in individuals over 60 years of age or those with platelet counts below 20–30 × 10^9^/L. Contributing factors to these clinical differences may include distinct immunologic triggers, comorbidities, comedications, and age-related immune senescence. Accordingly, adults with severe thrombocytopenia are generally advised to receive platelet-enhancing therapy, whereas a watch-and-wait strategy is recommended for children with minor bleeding (1, 2). These different approaches underscore the importance of age in ITP management; however, adolescents and young adults (AYAs) do not neatly fit into either the pediatric or adult ITP group. Because most large-scale ITP studies focus on children under 10 years or adults over 40 or 50, data on this intermediate age range are limited. As a result, current guidelines—largely based on preschool or older adult populations—frequently overlook AYAs, underscoring the need for a more nuanced, age-adapted approach.

**Figure 1:**
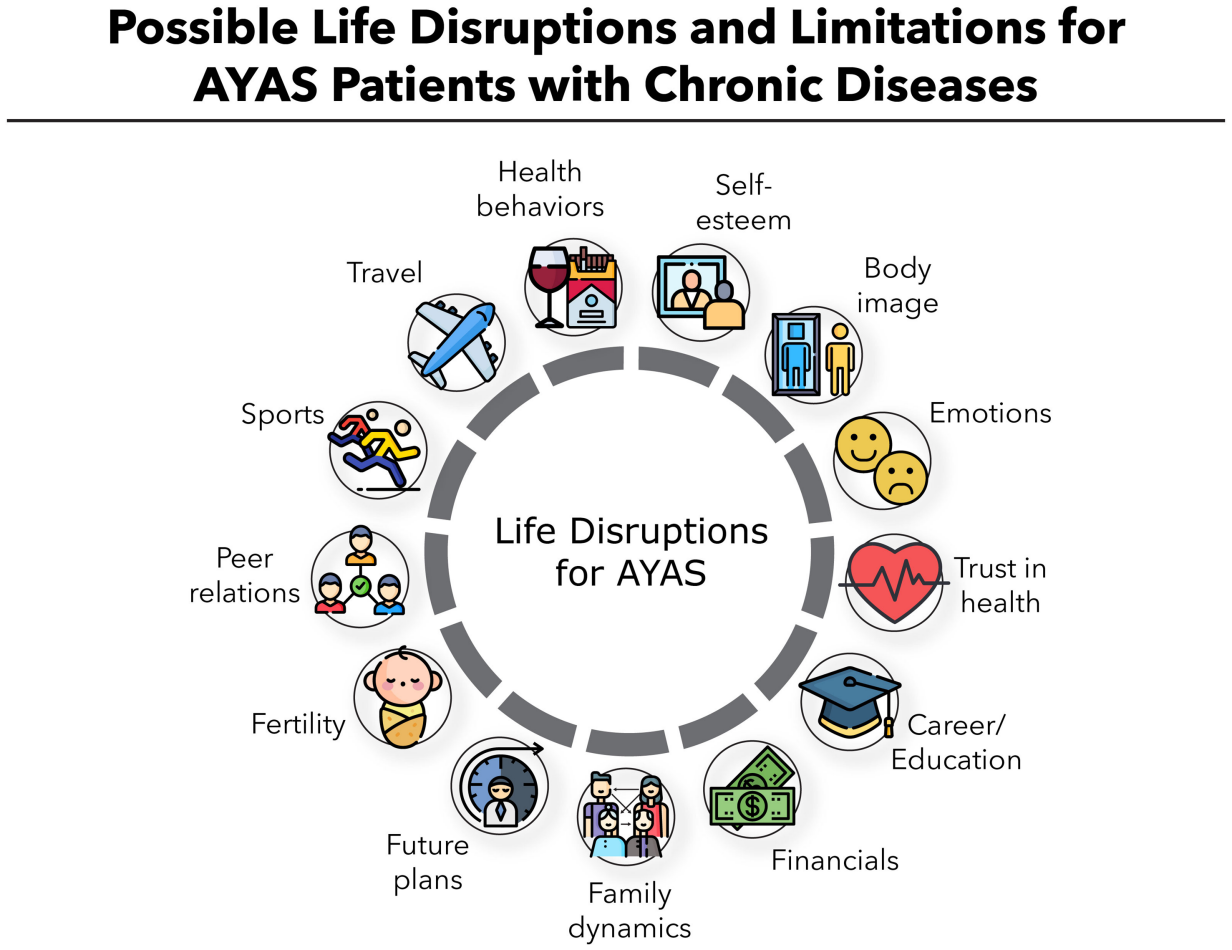
Possible life disruptions and limitations for AYAs patients with chronic ITP.

AYAs are increasingly recognized as a distinct patient population with different health and psychosocial characteristics and expectations. This was first demonstrated in oncology (3, 4). This life stage is marked by significant physiological, hormonal, and social changes. AYAs are often navigating critical school or career milestones, and experiencing evolving social networks. Chronic ITP can substantially disrupt these developmental tasks (Figure 1): limitations on physical activity, frequent medical appointments, and the psychological stress of unpredictable bleeding episodes can affect educational progress and social integration. Moreover, treatment may cause side effects, adherence challenges, or be contraindicated in women who wish to become pregnant. Repeated corticosteroid use can cause weight gain, mood disturbances, acne, or reduced bone density—all of which can negatively impact self-esteem and health-related quality of life (HRQoL).

Studies indicate that patients over 10 years of age have a greater tendency to develop chronic ITP (5–7). This suggests that AYAs may exist in a “border zone” between the high self-limiting potential observed in younger children and the pronounced tendency for chronic disease seen in older adults. However, biologically, it could be hypothesized that the still-maturing immune system in AYAs retains enough plasticity to reverse abnormal immunological processes and restore self-tolerance. This might offer higher chances of long-term remission, provided therapies are tailored to foster immunomodulation. Preliminary findings suggest that early, more intensive immunomodulation might reduce the risk of ITP becoming chronic (8–11). Although such an approach has not been sufficiently evaluated in AYAs (11), it could be particularly beneficial in this age group. AYAs with ITP therefore require treatment strategies that balance the higher risk of chronicity and disease burden against the potential for overtreatment and side effects. Clinicians should also consider how best to support AYAs’ needs and minimize the adverse psychosocial effects of both the disease and its treatment.

The AYAs collaboration—drawing on data from the *Pediatric and Adult Registry on Chronic ITP* (PARC-ITP), the *Cytopénies Auto-immunes Registre Midi-Pyrénéen-France* (CARMEN-France) adult registry in Toulouse, and the *National Prospective Cohort for Children with Chronic Autoimmune Cytopenia* (OBS’CEREVANCE) in Bordeaux, France—aims to address the information gap in AYAs with ITP. To date, four analyses have been undertaken (using data from 2004 to 2021), each addressing the major clinical aspects of ITP in patients aged 12–25 years: (1) newly diagnosed ITP (12), (2) chronic disease (13), (3) refractory courses (14), and (4) secondary (sITP) forms (15).

A deeper understanding of ITP’s natural history, risk factors for chronicity, and outcomes in AYAs is a crucial first step toward developing tailored treatment algorithms. Such data could form the basis for recommendations targeting this underrepresented yet clinically distinct population. Ultimately, age-adapted trials may improve long-term outcomes, reduce toxicity, and enhance HRQoL for AYAs with ITP.

## Discussion

### The first year of ITP

In this first analysis, we included 656 AYAs (mean age 15.3 years, SD 2.5; 61% female) with newly diagnosed ITP from the PARC and CARMEN registries. Overall, the AYA cohort was homogeneous regarding clinical characteristics, with no clear rationale to separate adolescents from young adults or males from females.

The analysis revealed that AYAs exhibit clinical features overlapping both pediatric and adult forms of ITP (Table 1). Similar to younger children, AYAs typically had few comorbid conditions, frequent bleeding (82%), and very low platelet counts (median 12 × 10^9^/L) at initial presentation. Yet, echoing the adult population, we observed a higher proportion of females (61%), a substantial likelihood (>50%) of chronic disease at 12 months, and a high use of corticosteroids throughout the one-year observation period, although dose and duration details were not captured. Possible reasons for persistent steroid use include the drug’s convenience, on-demand availability (e.g., for menstrual bleeding, preoperative treatment, rescue therapy), and limited or expensive access to alternatives [e.g., thrombopoietin receptor agonists (TPO-RAs)] during the study period.

**Table 1:** Clinical characteristics of newly diagnosed primary ITP in different age groups [adapted and expended from Schifferli et al. (12, 23)].

Patients with newly	Children (<16y)	AYAs (12-25y)	Adults (>16y)
diagnosed ITP	(Data of the PARC-Registry)	*N* = 656	(Data of the PARC-Registry)
Gender	46% female	61% female	68% female
Co-morbidities	6%–10%	15%	30%
Initial platelet <20 × 10^9^/L	79%	64%	58%
Bleeding initial	87%–90%	82%	70%
Bleeding type, initial	Skin 86%	Skin 72%	Skin 61%
	Epistaxis 20%	Epistaxis 21%	Epistaxis 16%
		Gynecological 22%	Gynecological 11%
ICH total (until 12 mo)	0.4%–0.6%	*n* = 4 (0.6%)	1.9%
Initial treatment	69%–80%	66%	67%–71%
Treatment between 6 and 12 months	19%	30%	40%

Our data confirm that older pediatric patients (>10 years) face a higher risk of chronic disease, consistent with earlier studies, though definitions of “remission” and “chronic disease” vary in the literature. For example, the ICIS I registry reported remission rates of 72% in children aged 1–10 but only 53% in those aged 10–16 (6). Likewise, in a U.S. cohort of 10- to 18-year-olds (1976–2000), more than half developed chronic disease (16). In our cohort, adolescents aged 15–18 had a slightly higher chronic disease rate (61%) than those aged 12–15 (55%), supporting the continuum of age-dependent remission rates. There were no sex-based differences in remission rates, consistent with earlier reports (16).

Individuals entering remission had initial severe thrombocytopenia (<20 × 10^9^/L) more frequently than those following a chronic course (69% vs. 56%). Conversely, 65% of patients with initially mild or moderate thrombocytopenia (≥20 × 10^9^/L) developed chronic ITP, mirroring patterns previously reported in both pediatric and adult ITP. Interestingly, AYAs with severe thrombocytopenia who received upfront treatment were less likely to develop chronic disease than those managed with watch-and-wait (47% vs. 68%, *p* < 0.05), suggesting that early immunomodulation or immunosuppression may induce lasting responses in AYAs with “pediatric-like” acute severe ITP. Conversely, frontline therapy in with initial moderate or mild thrombocytopenia correlated with lower remission rates (26%) than a watch-and-wait approach (41%). One explanation could be that patients experiencing bleeding despite moderate thrombocytopenia may have alternative causes of thrombocytopenia (e.g., hereditary or secondary ITP), making standard therapies less effective.

Debate continues over whether aggressive intervention early in the disease can prevent chronicity. Emerging protocols using combined T- and B-cell–directed approaches, sometimes with TPO-RAs, have shown encouraging outcomes in small trials (8–11). Early rituximab in young women may also help establish prolonged immune tolerance (17). Because AYAs often exhibit a clinical course more akin to adults, they may benefit from proactive measures aimed at deeper immunological remission rather than merely controlling bleeding. However, further refinement is needed, particularly around identifying which AYAs are at the highest risk for chronic or refractory disease.

### Chronic primary ITP

A total of 427 AYAs (64% female) with chronic primary ITP (pITP) from all three registries were included in this analysis. Overall, we observed steady clinical improvement up to 48 months of follow-up (FU) despite chronicity, evidenced by fewer bleeding events, higher mean platelet counts, and reduced overall treatment use. In total, 67 patients (16%) were managed with a watch-and-wait strategy until the last available FU (37 had complete data up to 48 months), while 167 (39%) received first-line therapies [corticosteroids, intravenous immunoglobulins (IVIG)] only. This finding aligns with pediatric data indicating that many children remain on first-line treatment alone—likely reflecting an on-demand approach (1, 18). In total, 188 (44%) patients received second-line drugs until the last available FU. Despite the increasing use of second-line agents over time among treated patients, the proportion receiving IVIG remained stable at about 40% across all FUs, possibly reflecting platelet fluctuations, breakthrough bleeding, or difficulties tapering treatment. Corticosteroids were administered to approximately 31–47% of treated patients, though dosage and duration were not reported. It is to note that TPO-RAs, licensed for pediatric ITP since 2016/2018, are underrepresented in our analysis, and treatment practices have evolved considerably since then.

Bleeding patterns stayed largely unchanged, with 60% of symptomatic patients presenting wet bleeding, but gynecologic bleeding rose as more girls entered puberty. Intracranial hemorrhage (ICH) was rare; six of eight events occurred before chronicity, aligning with studies indicating that ICH tends to appear early in the disease course and more frequently in older adults (≥60 years) (19–22).

Among the 427 patients with chronic ITP at 12 months, 99 (23%) achieved sustained complete remission off treatment (SCROT) during subsequent FUs, evenly spread over 3 years. SCROT was defined as platelet count >100 × 10^9^/L without treatment for at least 12 months, independently of the previous treatment strategy. This result likely underestimates true remission rates, as those lost to FU often have milder disease courses (23). Unfortunately, variations in chronic ITP definitions, remission criteria (platelet threshold and time off treatment), and study designs complicate direct comparisons with published data (24–31). Nonetheless, in our cohort, roughly 10% of chronic ITP patients per year attained SCROT, matching some pediatric findings (31).

Identifying predictors of remission is crucial for tailoring treatment and counseling patients. Previous publications show that factors such as older age (>10 years), higher initial platelet counts, or lack of severe bleeding are associated with chronic disease risk in all age categories (6, 16, 32–34), yet evidence for late remission predictors remains limited (7, 31, 35–37). In our study, AYAs who achieved “late SCROT” (12–36 months) tended to show a more favorable disease course during the first year (excluding initial presentation) than those with “ongoing chronic disease.” Indeed, these patients presented with higher platelet counts, less bleeding, and reduced IVIG use between 6 and 12 months. This pattern was not observed in AYAs who achieved “very late SCROT” (between 36 and 48 months).

Overall, although AYAs have a higher risk of chronic disease at 12 months than younger children, their late remission rates and treatment profiles resemble pediatric patterns (Table 2). Because a notable proportion eventually achieves SCROT beyond 1 year, clinicians should consider postponing splenectomy.

**Table 2:** Clinical characteristics of chronic primary ITP in different age groups [adapted and expended from Schifferli et al. (13, 23)].

Patients with chronic ITP	Children with chronic ITP	AYAs	Adults with
	(Data of the PARC-Registry)	*N* = 428	(Data of the PARC-Registry)
Chronicity	29%	50%	~ 70%
Bleeding, between 12 and 24 months	65%	49%	36%
Bleeding type, between 12 and 24 months	Skin 85%	Skin 77%	Skin 85%
	Skin only 47%	Skin only 34%	Skin only 46%
	Epistaxis 32%	Epistaxis 32%	Epistaxis 23%
		Gynecol. 48%	Gynecol. 20%
	ICH 0.6%	ICH *n* = 2 (0.5%)	ICH no cases *(published~1%)*
Treatment between 12 and 24 months	47%	47%	40%
Drug used (% of treated patients)	Steroid 59% IVIG 57% Second line 44%	Steroid 47% IVIG 40% Second-line 54%	Steroid 68% IVIG 26% Second-line 65%
Risk factors of chronicity	*Literature:* *Age > 10 years* *Sex unclear* *No preceding viral infection* *Initial mild thrombocytopenia*	Initial Tc >20 × 10^9^/L or Initial Tc <20 × 10^9^/L following an initial watch and wait strategy	*Literature: Initial mild thrombocytopenia*
Sustained complete remission off treatment (SCROT)	*Literature: 30–50%*	*n* = 99 (represents 10% new SCROT cases per year at 24, 36, and 48 months FU)	*No clear data in the literature*
Chronic refractory ITP	*No clear data in the literature*	*n* = 60, (14% of AYAs with chronic ITP, analyzed until 48 months FU)	*Literature:* ~ *10% (depending on definition)*

### Chronic refractory ITP

Only a small proportion (14%) of AYAs with chronic pITP in our cohort met our criteria for refractory disease, defined as the administration of ≥2 second-line treatments. Given that about half of AYAs develop chronic ITP, the overall proportion of newly diagnosed patients who will develop chronic refractory ITP is approximately 7%, which aligns with adult epidemiological estimates (~3%) (38). Median age at diagnosis was similar in both chronic refractory and chronic non-refractory groups, but the proportion of males was higher in the refractory group (43%) compared to the non-refractory group (35%). This difference was even greater among patients who were already refractory at 12 months (early refractory, *n* = 29, 48% male).

Patients with refractory disease had a notably higher clinical burden, including lower platelet counts, more frequent bleeding (especially wet bleeding), and greater ongoing treatment requirements compared to those with non-refractory ITP. In those identified as refractory early in the disease course, platelet counts and bleeding rates were even more severe at diagnosis and during the first year of FU, consistent with the literature (39). The proportion of patients with initial platelet counts <20 × 10^9^/L was 57% in the non-refractory group, 64% in the refractory group, and 74% in the early refractory subgroup. Findings from the French CARMEN registry of adults with multirefractory ITP similarly showed very low initial platelet counts (6 × 10^9^/L) and a high early bleeding rate (88%) (38). Likewise, a 2016 multicenter study by Mahévas et al. (40) on 37 multirefractory patients compared with a historical ITP cohort (*n* = 183) identified lower baseline platelet counts (median 9 × 10^9^/L vs. 17 × 10^9^/L) and higher bleeding/infection-related morbidity and mortality in the refractory group, although no significant gender differences emerged.

Despite the extensive use of multiline therapies at all FU points in our study, about 25% of AYAs with refractory disease continued to rely on corticosteroids at last FU (48 months), compared with only 10% of non-refractory patients. This persistent steroid use remains concerning, given that long-term or excessive steroid therapy is the most common toxicity in ITP. Corticosteroids often serve as an effective add-on or on-demand option for menstruating women, pre-procedural settings, travel, or emergencies. However, access to TPO-receptor agonists was limited in the study period, particularly for children under 16.

Overall, refractory ITP in AYAs appears to present with more severe disease features early on, implying a potentially distinct disease biology rather than just a gradual progression. However, the pathogenesis remains poorly understood and various hypotheses exists supporting progression (epitope spreading, increased drug-efflux pumps, transition from antibody- to T-cell–mediated autoimmunity, oligoclonal or monoclonal T-cell receptor expansions, expansion of long-lived plasma cells) (41–47), and supporting distinct diseases [different etiopathologies or antigen targets (48, 49), misdiagnoses like hereditary thrombocytopenia, bone marrow failure, or sITP (39, 40)]. Mahévas et al. (40) noted that 35% of multirefractory cases had sITP, versus only 9% in the control group. This heterogeneity underscores the challenges of labeling patients as “refractory” or “difficult to treat,” since they comprise a highly diverse subgroup.

Our definition of refractory ITP can also influence its comparability with other studies. Existing guidelines (50) define refractory disease as not responding to splenectomy, a criterion less applicable to adolescents, for whom splenectomy is often delayed. Although new studies have expanded the criteria, they are frequently too stringent (40), and often fail to capture the “difficult-to-treat” patients that clinicians encounter in routine practice. Our pragmatic approach—defining refractory disease as requiring ≥2 second-line therapies—focuses on a small group with hard-to-manage ITP and is easy to apply in both clinical and research settings. Strengthening the definition further would significantly reduce the number of identified patients, thereby limiting the potential for useful data analysis.

In conclusion, although refractory ITP in AYAs is relatively infrequent, the disease burden is high. Individuals most likely to follow a refractory course are often male, present with very low platelet counts at diagnosis, and experience repeated wet bleeding in the first year. However, these early distinctions alone do not reliably predict long-term outcomes or justify aggressive early intervention or study enrollment. Hence, new early markers of refractoriness are needed to refine patient stratification, and guide treatment strategies in AYAs.

### Chronic secondary ITP

**Figure 2:**
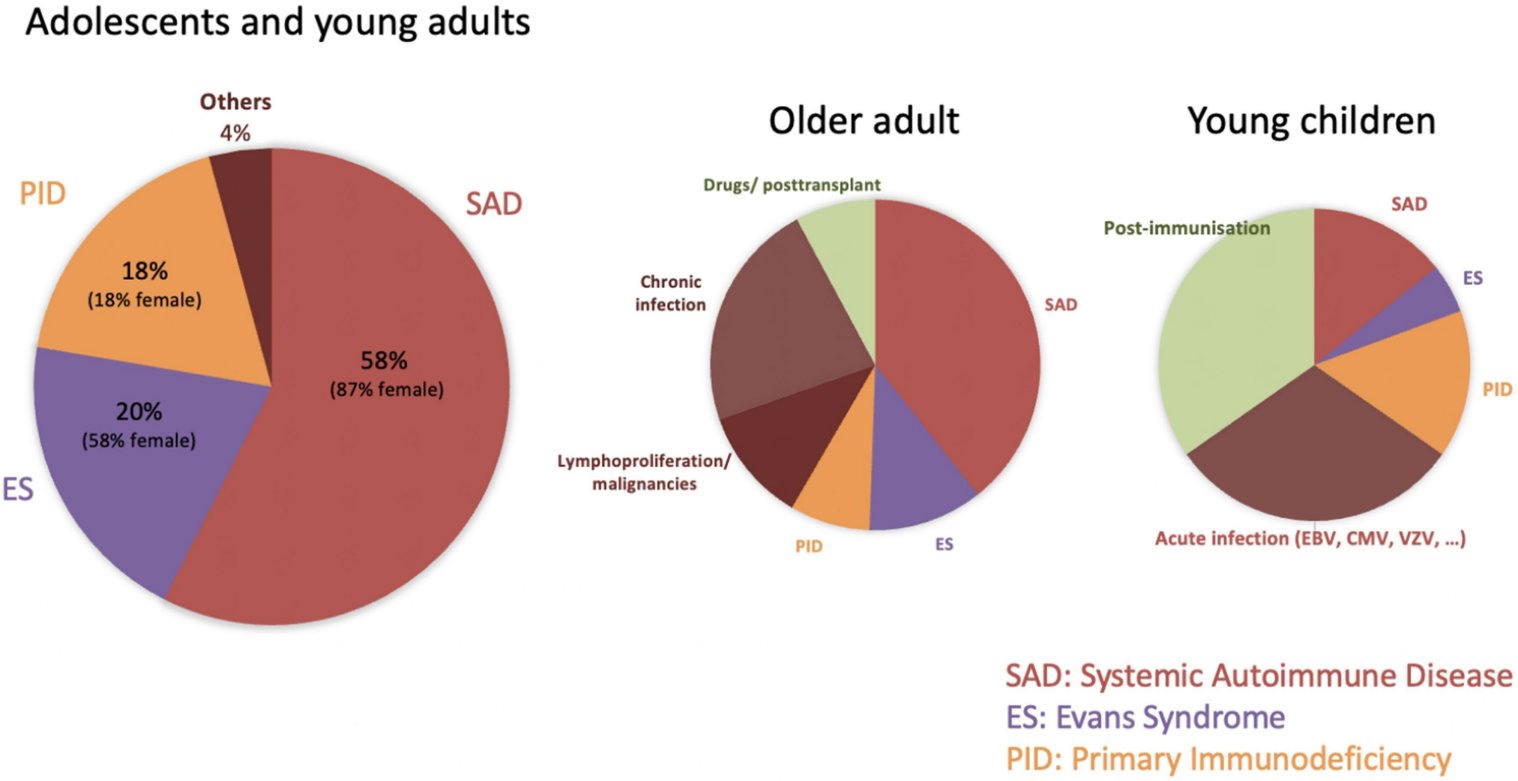
Causes of secondary ITP in different age groups.

Among 481 AYAs with chronic disease from all three registries, 94 had an identifiable underlying condition (sITP), representing 20% of chronic cases and 10% of all newly diagnosed ITP. This rate lies between pediatric (3%) and adult (20%) estimates, confirming that sITP becomes more common with age. In our cohort, systemic autoimmune disorders (SAD) accounted for the largest proportion of sITP (57%), followed by Evans syndrome (ES, 20%) and primary immunodeficiencies (PID, 18%) (Figure 2). These subgroups comprise therefore ~ 11% (SAD), 4% (ES), and 3.5% (PID) of all chronic ITP. Existing studies on sITP often mix acute and chronic patients, making direct comparisons difficult. Nonetheless, the prominence of SAD conditions—particularly systemic lupus erythematosus (SLE)—aligns with previous reports, given that about one-third of SLE patients develop thrombocytopenia, and 2–5% of adults with ITP are later diagnosed with SLE.

Marked gender imbalances emerged among sITP subgroups, with 87% of SAD cases being female (reflecting the known female predominance in autoimmune disorders) and 82% of PID cases being male. Despite these differences, initial disease features (platelet count, bleeding incidence and types, and treatment need) were similar across the three sITP subgroups and the chronic pITP cohort.

Only 27% of sITP cases were recognized at ITP onset, whereas 30% were diagnosed more than 2 years later. Delayed diagnoses were particularly common in PID. These findings underscore the need for ongoing re-evaluation of chronic ITP, consistent with adult studies that report about 12% of presumed pITP cases are reclassified during FU (51). Current guidelines support systematic screening when new symptoms or laboratory abnormalities arise (52), and recent publications emphasize the utility of genetic testing in pediatric ITP with atypical (chronic or refractory) presentations (53, 54). Although disease-related genetic findings in ITP are rare, they can guide patient counseling and targeted therapies. Additionally, broader use of genetic diagnostics will gradually improve our understanding of immune dysregulation. In Evans syndrome, 39–65% of children exhibit PID-related gene variants, underscoring the importance of genetic counseling in multiple cytopenias (55, 56).

Based on our observations (Figure 2), we recommend biannual assessments for AYAs with persistent or chronic ITP, comprising a thorough clinical evaluation, complete blood count with reticulocytes and blood smear review, and Coombs testing if indicated. For females, screening for autoimmune markers (e.g., ANA) and systemic autoimmune symptoms is warranted, while immunodeficiency screening (e.g., immunoglobulin levels) should be a priority for males. These measures can help detect secondary causes of ITP earlier, facilitating timely and individualized management.

## Conclusion

Collectively, our four analyses demonstrate that AYAs with ITP do not fit neatly into traditional “pediatric” or “adult” categories. The initial presentation of ITP in AYAs resembles pediatric ITP (abrupt, severe, symptomatic thrombocytopenia with very low risk of ICH), but the disease course resembles adult ITP. However, AYAs have fewer comorbidities and secondary forms and may retain a greater potential for late remission compared to older adults. Although most AYAs with chronic ITP maintain relatively stable courses over time, a subset progresses to refractory or sITP, each with distinct clinical and therapeutic challenges.

With their longer life expectancies and higher HRQoL standards, AYAs require new approaches that focus on sustainable immunomodulation rather than continuous, symptom-driven management. Future studies should therefore:

1. *Clearly define AYAs as a distinct patient subgroup* rather than combining them with either pediatric or adult cohorts.
2. *Refine treatment goals* to reduce long-term chronicity, including earlier, targeted immunomodulation when appropriate.
3. *Discourage extended corticosteroid use* beyond the acute phase to minimize toxicity and preserve HRQoL.
4. *Improve recognition and workups for secondary ITP*, enabling timely, individualized care for those with underlying autoimmune disorders or immunodeficiencies.
5. *Address quality of life* in AYAs’ care: given the multitude of developmental and social challenges at this life stage, it is crucial to look beyond ITP symptoms and treatments alone, as the disease can negatively affect diverse aspects of their lives (Figure 1) (57–59).
